# Specific contact resistivity reduction in amorphous IGZO thin-film transistors through a TiN/IGTO heterogeneous interlayer

**DOI:** 10.1038/s41598-024-61837-2

**Published:** 2024-05-13

**Authors:** Joo Hee Jeong, Seung Wan Seo, Dongseon Kim, Seong Hun Yoon, Seung Hee Lee, Bong Jin Kuh, Taikyu Kim, Jae Kyeong Jeong

**Affiliations:** 1 Department of Electronic Engineering, Hanyang University, Seoul, 04763 Republic of Korea; 2 Semiconductor R&D Center, Samsung Electronics Co., Hwaseong, Gyeonggi 18848 Republic of Korea; 3 Electronic Materials Research Center, Korea Institute of Science and Technology, Seoul, 02792 Republic of Korea

**Keywords:** Electrical and electronic engineering, Electronic devices

## Abstract

Oxide semiconductors have gained significant attention in electronic device industry due to their high potential for emerging thin-film transistor (TFT) applications. However, electrical contact properties such as specific contact resistivity (*ρ*_C_) and width-normalized contact resistance (*R*_C_*W*) are significantly inferior in oxide TFTs compared to conventional silicon metal oxide semiconductor field-effect transistors. In this study, a multi-stack interlayer (IL) consisting of titanium nitride (TiN) and indium-gallium-tin-oxide (IGTO) is inserted between source/drain electrodes and amorphous indium-gallium-zinc-oxide (IGZO). The TiN is introduced to increase conductivity of the underlying layer, while IGTO acts as an n^+^-layer. Our findings reveal IGTO thickness (*t*_IGTO_)-dependent electrical contact properties of IGZO TFT, where *ρ*_C_ and *R*_C_*W* decrease as *t*_IGTO_ increases to 8 nm. However, at *t*_IGTO_ > 8 nm, they increase mainly due to IGTO crystallization-induced contact interface aggravation. Consequently, the IGZO TFTs with a TiN/IGTO (3/8 nm) IL reveal the lowest *ρ*_C_ and *R*_C_*W* of 9.0 × 10^−6^ Ω·cm^2^ and 0.7 Ω·cm, significantly lower than 8.0 × 10^−4^ Ω·cm^2^ and 6.9 Ω·cm in the TFTs without the IL, respectively. This improved electrical contact properties increases field-effect mobility from 39.9 to 45.0 cm^2^/Vs. This study demonstrates the effectiveness of this multi-stack IL approach in oxide TFTs.

## Introduction

In 2004, amorphous indium-gallium-zinc-oxide (*a*-IGZO) was discovered by the group of Prof. Hosono, offering numerous outstanding characteristics such as reasonable field-effect mobility (*μ*_FE_) > 10 cm^2^/Vs, extremely low off-current < 10^−24^ A/μm, steep subthreshold swing (*SS*) of ~ 0.1 V/dec, and outstanding uniformity even when fabricated at low temperature^1–5^. Because of these merits, oxide semiconductor (OS) family has been studied intensively and has become a standard channel material of thin-film transistors (TFTs) in high-end active matrix organic light emitting diode (AMOLED) display backplanes. Recently, the OS has gained more attention as a channel candidate for next-generation semiconductor device applications such as 2T0C dynamic random access memory (DRAM)^6–9^, because it has potential to overcome the scaling and leakage problems of DRAM technologies due to their ultralow off-current originating from the wide bandgap (*E*_G_) nature. Furthermore, their back-end-of-line (BEOL)-compatible low-temperature processibility enables movement of the DRAM peripheral circuitry under the memory array, achieving 3D DRAM technology.

Solid state devices have been scaled down to improve power, performance, area, and cost (PPAC) in the semiconductor industry^2^. O/S TFTs also must follow this conventional PPAC rule to meet the abovementioned requirements. For this reason, increasing numbers of studies on scaled oxide TFTs have been reported^2^. Here, it is important to note that the impact of contact resistance in nanoscale semiconductor devices increases considerably with miniaturization. However, the contact resistance of OS TFTs is generally four orders of magnitude higher than that of the current silicon (Si) metal oxide semiconductor field-effect transistors (MOSFETs)^10–12^. This high contact resistance of OS TFTs can be a critical obstacle for miniaturized device applications^13^. Thus, there is need to improve the electrical contact properties between OS channels and source/drain (S/D) electrodes.

To improve the contact resistance at the channel/electrode interface, both Schottky barrier height and width must be reduced to facilitate carrier injection through the barrier formed at the interface^14–16^. The height is controlled depending on a work function of contact electrode, which can reduce the contact resistance^17^. The interface-state defects at the channel/electrode interface should be minimized to increase the height control^18^. The width determined by carrier density (*n*_c_) of the channel material can be narrowed by several approaches^19–33^. Increasing *n*_c_ is the simplest method to reduce barrier width, which was originally devised for the Si MOSFETs, and can be realized through ion implantation and/or plasma treatments using elements such as boron, fluorine, argon, or hydrogen^19–21,29,30^. However, these methods can damage the channel layer below the S/D electrodes, which increases the interface defects and offsets the merits of increasing *n*_c_. In addition to these doping techniques, other approaches such as controlling cation composition, metal-induced oxygen scavenging and highly conductive interlayer (IL) insertion have been employed^22–27,34,35^. These methods have an advantage in that they can reduce the barrier width without incurring damage.

In this study, a multi-stack IL using titanium nitride (TiN) and indium-gallium-tin-oxide (IGTO) is inserted between IGZO channel and indium tin oxide (ITO) S/D electrodes. Specific contact resistivity (*ρ*_C_) of IGZO TFTs with a 3-nm-thick TiN IL is reduced to 5.6 × 10^−5^ Ω·cm^2^, which is almost 20-fold lower than that of IGZO TFTs without the IL. More importantly, the electrical contact properties have IGTO thickness (*t*_IGTO_)-dependent behavior, decreasing with *t*_IGTO_ up to 8 nm. It is degraded in the IGZO TFTs with *t*_IGTO_ greater than 8 nm. Consequently, the device with a TiN/IGTO (3/8 nm) IL has the lowest *ρ*_C_ and width-normalized contact resistance (*R*_C_*W*) of 9.0 × 10^−6^ Ω·cm^2^ and 0.7 Ω·cm, which also leads to the largest increase in *μ*_FE_ from 39.9 to 45.0 cm^2^/Vs compared to the device without the IL. This noticeable improvement in the electrical contact properties could be attributed to enhanced electron injection by reduced Schottky barrier height (SBH) and width through the TiN/IGTO IL insertion, which will be discussed in depth.

## Methods

### Device fabrication

**Figure 1:**
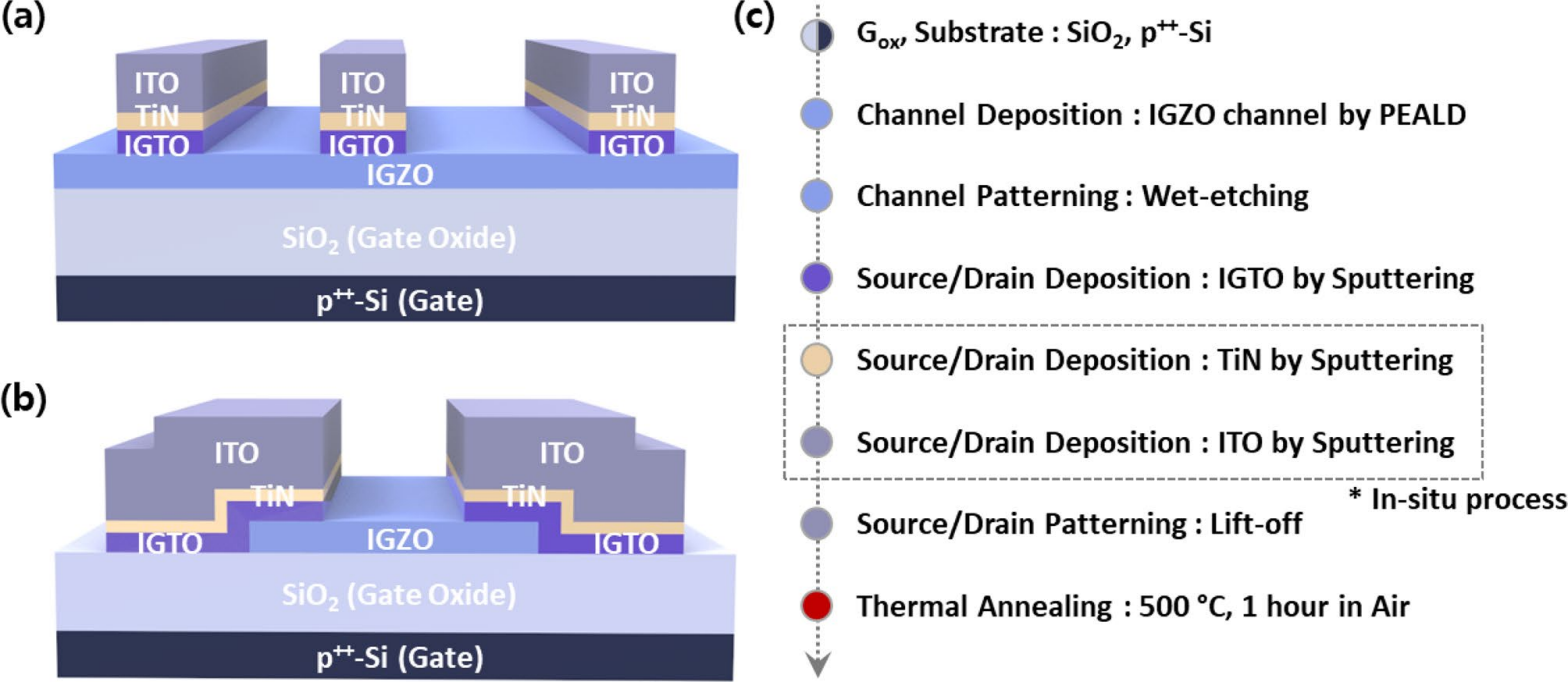
Schematic structures of the fabricated devices: (**a**) The TLM device; (**b**) the IGZO TFT; (**c**) device fabrication procedure.

Transmission line method (TLM) patterns and bottom gate IGZO TFTs were fabricated with top-contact configuration (Fig. 1a,b). 15-nm-thick IGZO thin-films were deposited through plasma-enhanced atomic layer deposition (PEALD) using dimethylbutylamino(trimethylindium) (DATI), trimethylgallium (TMG), diethylzinc (DEZ), and O_2_ plasma as In, Ga, Zn and oxygen sources, respectively, at 150 °C. Also, 100-nm-thick silicon oxide (SiO_2_) and heavily doped p^+^-Si substrates were used as gate dielectric and electrode, respectively. The deposited channel layers were patterned by standard photolithography and wet etching. The TiN and IGTO IL were deposited through direct current (DC) magnetron sputtering at room temperature, followed by in-situ deposition of highly conducting indium tin oxide (ITO) thin-films for S/D electrodes. Then, the deposited S/D structure with the multi-stack IL was patterned using the lift-off method. Finally, the devices were annealed at 500 °C under ambient air for 1 h. The fabricated TLM pattern devices have a channel width (*W*) of 60 μm and lengths (*L*) of 20, 30, 40, and 50 μm. The TFTs has *W*/*L* of 60/30 μm.

### Materials and device characterization

The film thickness was determined through spectroscopic ellipsometry (Elli-SE, Ellipso Technology), and *n*_c_ was obtained through Hall effect measurements (HMS-5000, Ecopia) with a van der Pauw configuration. Surface morphologies of IGTO/IGZO thin-film stacks were characterized using atomic force microscopy (XE-100, Park System Co.). The crystalline structure of the IGTO thin-film was confirmed through grazing incidence X-ray diffraction (Model X’Pert PRO MRD, Malvern Panalytical) and high-resolution transmission electron microscopy (NEOARM, JEOL). Chemical states were determined through X-ray photoelectron spectroscopy (K-Alpha+, Thermo Fisher Scientific Co.) after in-situ surface (~ 3 nm) etching using an accelerated (1 kV) Ar^+^ ion to avoid confusion by possible contamination and/or oxidation during air exposure.

Electrical characteristics of the fabricated TFTs were measured through a Keithley 2636 source meter at room temperature under dark conditions. The *μ*_FE_ was calculated using the equation ${\mu_{FE}} = \frac{{L{g_m}}}{{W{C_{OX}}{V_{DS}}}}$^31^, where *C*_OX_, *V*_DS_, and *g*_m_ are the capacitance per unit area, drain-to-source voltage, and transconductance, respectively. In this study, *V*_DS_ is 0.1 V. Threshold voltage (*V*_TH_) was extracted using the constant current method, which was defined as the gate-to-source voltage (*V*_GS_) inducing a drain current (*I*_D_) of *L*/*W* × 10 nA^31^. Subthreshold swing (*SS*) was obtained using the equation $SS = \frac{{d{V_{GS}}}}{{d\log {I_D}}}$ at the subthreshold region of transfer characteristics^31^.

## Results and discussion

**Figure 2:**
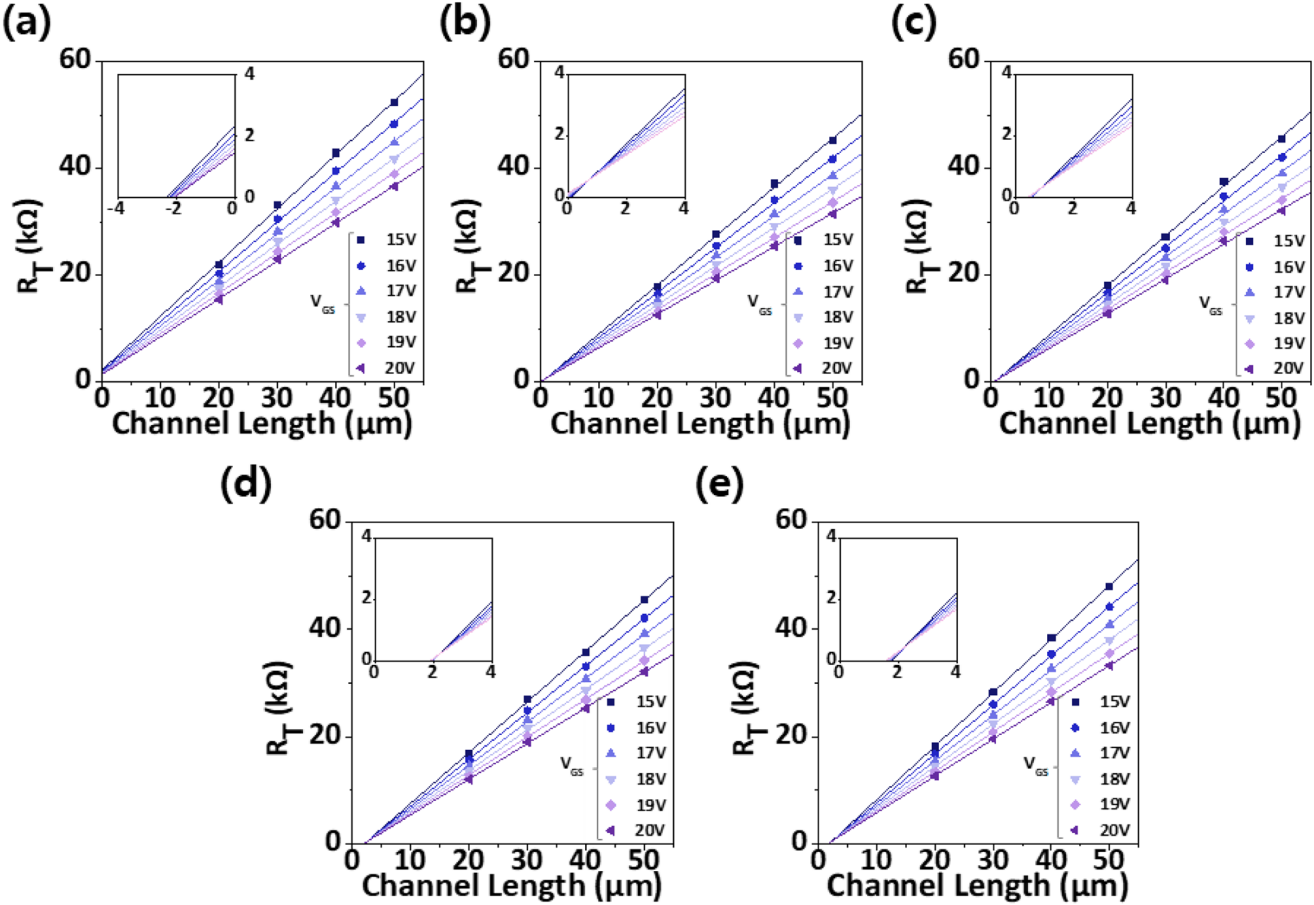
V_GS_-dependent R_T_ variations of the IGZO TLM devices with different multi-stack IL at V_DS_ of 0.1 V: (**a**) Without IL; (**b**) 3-nm-thick TiN IL; (**c**) 3-/5-nm-thick TiN/IGTO IL; (**d**) 3-/8-nm-thick TiN/IGTO IL; (**e**) 3-/12-nm-thick TiN/IGTO IL.

To verify the effects of TiN and IGTO IL on electrical contact properties in the IGZO TFT, TLM was conducted. Figure 2 shows total resistance (*R*_T_) versus *L* graphs as a function of gate voltage (*V*_GS_) in the IGZO TLM devices with different multi-stack ILs. *ρ*_C_ was calculated using the following equations:^14^

1 $$  {R_T} = {R_{ch}} + 2{R_C} = \frac{L - 2\Delta L}{{W{\mu_{FE}}{C_{OX}}\left( {{V_{GS}} - {V_{TH}}} \right)}} + 2{R_C} $$

where *R*_ch_, *R*_C_, and *∆L* are the channel resistance, the contact resistance, and the change in channel length, respectively. The values of 2*R*_C_ and 2*∆L* can be obtained at the intersection of the *R*_T_ and* L* curves (Fig. 2)^31^. More importantly, the *R*_C_*W* can be expressed as^36^

2 $$  {R_C}W = \sqrt {{\rho_C}{R_{SH}}} \;\coth \left( {\frac{{L_C}}{{L_T}}} \right) \approx \sqrt {{\rho_C}{R_{SH}}} ,\;\;{\text{if}}\;\;\;{L_C} \gg {L_T} $$

where *R*_SH_, *L*_C_, and *L*_T_ are the sheet resistance of the channel, the physical contact length (30 μm in this study), and the current transfer length, respectively. This equation can be rewritten as

3 $$  {\rho_C} = \frac{{{{\left( {{R_C}W} \right)}^2}}}{{{R_{sh}}}} $$

As such, *ρ*_C_ can be obtained using Eq. (3). It is also important to note that the *R*_C_*W*, an intuitive figure of merit to demonstrate the electrical contact properties, is simultaneously observed along with the *ρ*_C_ throughout this study. The control device without the IL has the largest *ρ*_C_ (*R*_C_*W*) of 8.0 × 10^−4^ Ω·cm^2^ (6.9 Ω·cm). Insertion of a 3-nm-thick TiN IL reduces this to 5.6 × 10^−5^ Ω·cm^2^ (1.8 Ω·cm), respectively, which indicates that the TiN IL improves the electrical contact properties. This improvement could be attributed to increase in conductivity of the underlying layer through the oxygen scavenging effect of the TiN. Combination of IGTO thin-film with the TiN IL further improves the electrical contact. Of particular interest is *t*_IGTO_-dependent behavior, where *ρ*_C_ (*R*_C_*W*) values of 1.4 × 10^−5^ (0.9), 9.0 × 10^−6^ (0.7), and 3.0 × 10^−5^ Ω·cm^2^ (1.3 Ω·cm) were obtained in the devices using a TiN/IGTO multi-stack IL with *t*_IGTO_ of 5, 8, and 12 nm, respectively. The device with the TiN/IGTO IL (3/8 nm) has the lowest *ρ*_C_ of 9.0 × 10^−6^ Ω·cm^2^, which is almost two orders of magnitude lower than that of the control device (Fig. S1). The *R*_C_*W* also has the same *t*_IGTO_ dependence which has the smallest value of 0.7 Ω·cm. It is noteworthy that the both of the *ρ*_C_ and *R*_C_*W* are reduced to 1.1 × 10^−5^ Ω·cm^2^ and 1.0 Ω·cm in the device featuring an 8-nm-thick IGTO IL (Fig. S2). This outcome suggest that the IGTO IL alone can enhance the electrical contact properties to a certain degree, even without the presence of TiN. However, it simultaneously indicates that the TiN/IGTO multi-stack IL holds greater influence. Additionally, it is worth noting that the device annealed at 400 °C with the TiN/IGTO IL (3/8 nm) demonstrates electrical contact properties comparable to those of the device with the same IL stack annealed at 500 °C (Fig. S3). These findings collectively imply that insertion of a multi-stack IL is not only an effective but also thermally stable method for enhancing the electrical contact properties of IGZO TFT.

Before comprehending the effect of *t*_IGTO_ on the electrical contact properties, it is quite important to discuss the implications of the intersection in *R*_T_ versus *L* graphs. In oxide TFTs, the *∆L* generally originates from oxygen vacancy (*V*_O_)’s diffusion, which occurs more noticeably in the OS containing high *V*_O_ concentration. Here, IGZO TFTs with an IL can have higher *V*_O_ concentration at the contact region than those without an IL due to a synergetic effect of TiN and IGTO layers where the former induces oxygen scavenging effect from the underlying oxide layer and the latter has high *V*_O_ concentration. It leads to the higher *n*_c_, which reduces the Schottky barrier width and improves the electron injection from the source electrode. More importantly, this reduced barrier width makes the electron injection independent on *V*_GS_. For this reason, the IGZO TFTs with an IL can have a *V*_GS_-independent intersection, i.e., *V*_GS_-independent *R*_C_, in the *R*_T_ versus *L* graphs. Meanwhile, there could not be an intersection, as shown in the *R*_T_ versus *L* graph of IGZO TFTs without an IL. In this case, the *R*_C_ as well as *L*_T_ becomes dependent on the *V*_GS_. It could be because the *n*_c_ under the S/D electrode is affected by the *V*_GS_ due to the relatively low *V*_O_ concentration at the contact region.

**Figure 3:**
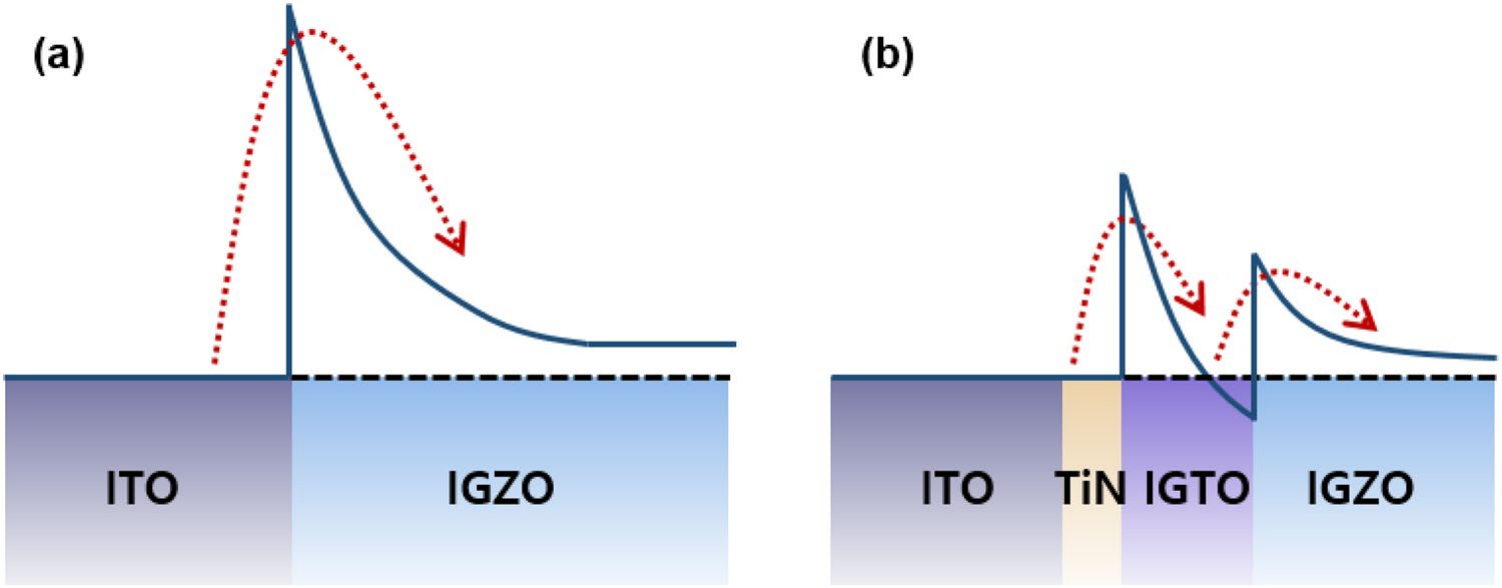
Schematic energy band diagrams at the contact region depicted based on UPS and UV/visible analyses: (**a**) without the IL; (**b**) 3-/8-nm-thick TiN/IGTO IL.

To understand the effect of multi-stack IL, the ultraviolet photoelectron spectroscopy (UPS) depth profile and UV/visible spectroscopy was conducted for two cases: (1) the ITO/IGZO thin-film stack; (2) ITO/TiN/IGTO/IGZO (3-/8-nm-thick TiN/IGTO IL) thin-film stack. Work functions and differences between the Fermi-level and the valence band edge (*E*_F_–*E*_V_) were obtained by the UPS (Fig. S4). *E*_G_ was extracted through the UV/visible spectroscopy. It was confirmed that both of the IGTO and IGZO possess approximately 3.6 eV of *E*_G_. Utilizing the values obtained from these analyses, the energy band diagrams were estimated (Fig. 3). The detailed procedure to depict the band diagrams can be seen in the previous study^37^. Consequently, the SBH decreases from approximately 0.4 to 0.2 eV by inserting the 3-/8-nm-thick TiN/IGTO IL. These values may deviate slightly. However, it is obvious that the SBH for electron injection is drastically reduced by this contact scheme. This reduction can lead to the improvement in specific contact resistivity by enhancing the electron injection as confirmed in the TLM analyses.

**Figure 4:**
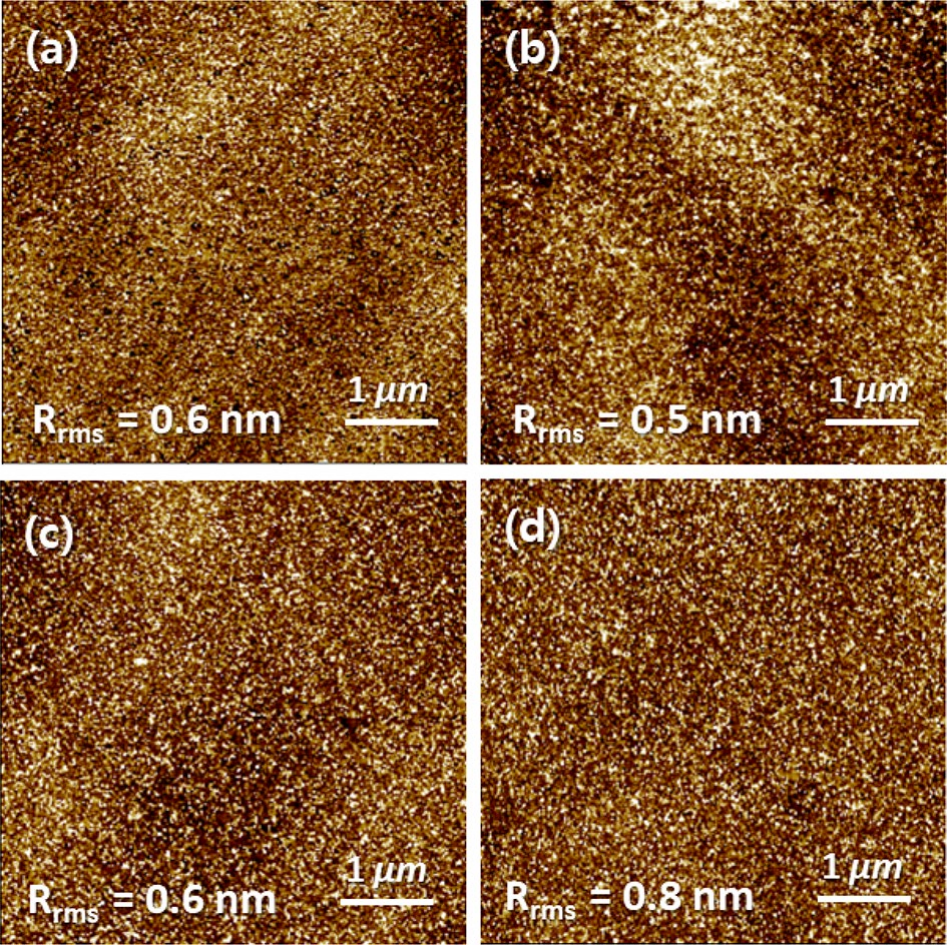
Surface images of the IGTO/IGZO thin-film stacks with different *t*_IGTO_: (**a**) 0 nm; (**b**) 5 nm; (**c**) 8 nm; (**d**) 12 nm.

**Figure 5:**
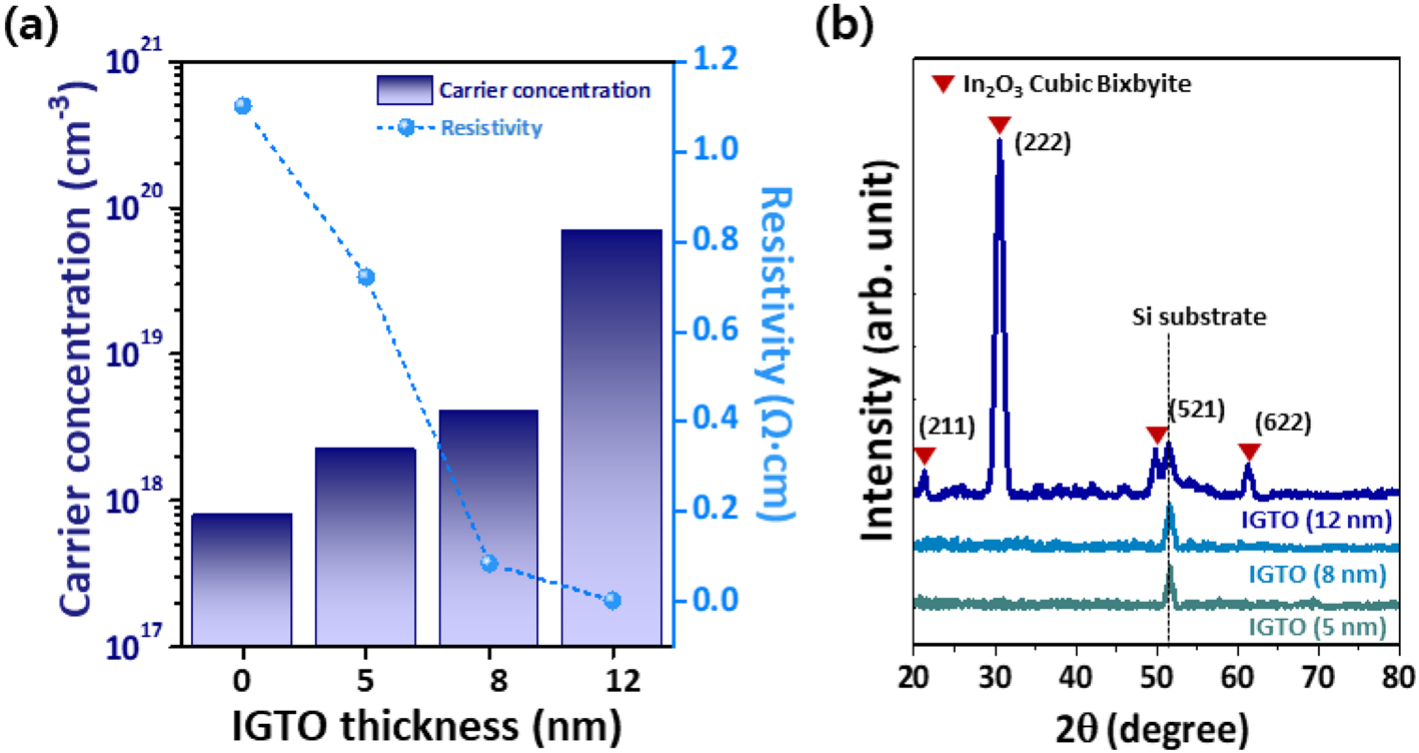
(**a**) Hall effect measurements of the IGTO/IGZO thin-film stacks with different *t*_IGTO_: 0, 5, 8, and 12 nm. (**b**) GIXRD patterns of the IGTO thin-films with different *t*_IGTO_: 5, 8, and 12 nm.

Then, atomic force microscopy (AFM) analysis was conducted. Figure 4 shows *t*_IGTO_-dependent surface roughness (*R*_rms_) of the IGTO thin-films deposited on the 15-nm-thick IGZO/SiO_2_/Si substrates. The *R*_rms_ increases to 0.8 nm in the 12-nm-thick IGTO thin-film, and this roughest surface could partially contribute to degradation of the electrical contact properties. The data obtained from the 0-nm-thick IGTO thin-film indicates information of the underlying 15-nm-thick IGZO thin-film. In addition to AFM analysis, Hall effect measurement was performed on the IGTO thin-films to investigate the origin of the *t*_IGTO_-dependent the electrical contact. The *n*_c_ of the IGTO thin-film steadily increases from 7.9 × 10^17^ to 6.9 × 10^19^/cm^3^ with *t*_IGTO_ (Fig. 5a). Moreover, its bulk resistivity (*ρ*_IGTO_) simultaneously decreases from 1.1 Ω·cm to 3.7 × 10^−3^ Ω·cm, a trend originating from percolation conduction^2^. This result shows that the IGTO IL can act as an n^+^-layer in IGZO devices but cannot elucidate the *t*_IGTO_-dependent the electrical contact. Even, the fact that the 12-nm-thick IGTO thin-film has the lowest *ρ*_IGTO_ conflicts with the trend that the *ρ*_C_ and *R*_C_*W* are the lowest in the *t*_IGTO_ of 8 nm.

Grazing-incidence X-ray diffraction (GIXRD) was conducted to investigate the crystalline structure of the IGTO thin-film (Fig. 5b). Surprisingly, the 12-nm-thick IGTO thin-film possesses a random polycrystalline In_2_O_3_ cubic bixbyite configuration, differing from the other *t*_IGTO_ conditions. Electron transport can be enhanced by decreased defect scattering after crystallization due to structural ordering and defect confinement, which can contribute to the decrease in bulk *ρ*_IGTO_ observed in the Hall measurement. More importantly, this crystallographic change has a large impact on a metal/semiconductor (MS) interface, a critical factor in the electrical contact properties, and can result in a trend difference between the electrical contact and *ρ*_IGTO_. However, it is possible that the IGTO thin-films with *t*_IGTO_ < 12 nm were crystallized but too thin to determine their crystalline structure using GIXRD.

**Figure 6:**
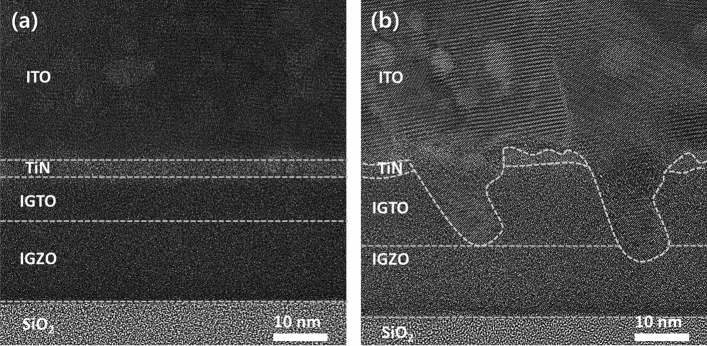
Cross-sectional HRTEM images of ITO/TiN/IGTO/IGZO stacks with different *t*_IGTO_: (**a**) 8 nm; (**b**) 12 nm.

For this reason, cross-sectional high-resolution transmission electron microscopy (HRTEM) was performed on the ITO/TiN/IGTO/IGZO stacks to clearly examine the *t*_IGTO_-dependent crystallographic change (Fig. 6). It is noteworthy that electron dispersive spectroscopy (EDS) depth profile was conducted to distinguish the IGTO and IGZO layers (Fig. S5). There is no noticeable nano-/microscale crystal in the 8-nm-thick IGTO thin-film (Fig. 6a and Fig. S6). The fast Fourier transform (FFT) result also shows a diffused hollow ring pattern (Fig. S7). These results indicate that the corresponding IGTO thin-film has an amorphous structure. Meanwhile, the 12-nm-thick IGTO thin-film is crystallized (Fig. S8), and many crystal grains significantly infiltrate from the ITO into the IGTO film (Fig. 6b and Fig. S8). It is worth mentioning that the ITO thin-film has the same In_2_O_3_ cubic bixbyite crystalline configuration. Consequently, the MS interface is considerably deteriorated in the thin-film stack with *t*_IGTO_ of 12 nm. These degradations are not observed in the stack with *t*_IGTO_ of 8 nm (Fig. S6). This deteriorated interface could be attributed to the worse thermal stability of the polycrystalline structure with grain boundaries than the amorphous structure that make it susceptible to thermal stress during annealing. Thus, the polycrystalline 12-nm-thick IGTO thin-film could be more prone to deformation and cracking by thermal stress compared to the amorphous 8-nm-thick IGTO thin-film, as observed in the HRTEM analyses. Here, it is important to note that the interfacial property can significantly influence electrical contact properties. This is because a rough interface can introduce numerous scattering and localized trapping centers, thereby impeding electron injection at the MS contact region. Furthermore, such roughness can induce variations in the Schottky barrier height, resulting in fluctuations in the contact properties. Consequently, this TEM result implies that the electrical contact can be greatly degraded in a device using the multi-stack IL with *t*_IGTO_ of 12 nm due to crystallization-induced disruptive interface even if it has the lowest bulk *ρ*_IGTO_. This underscores the critical importance of the interfacial quality between the S/D electrodes and the channel layer.

**Figure 7:**
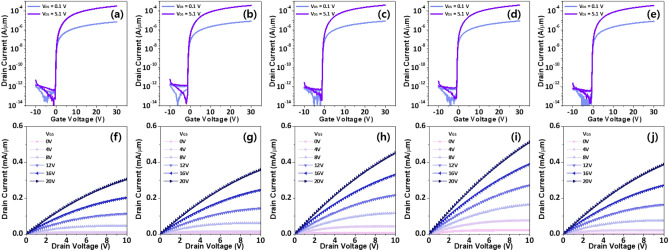
(**a**–**e**) Transfer characteristics of IGZO TFTs with different IL stacks: (**a**) Without IL; (**b**) TiN IL; (**c**) TiN/IGTO IL (*t*_IGTO_ = 5 nm); (**d**) TiN/IGTO IL (*t*_IGTO_ = 8 nm); (**e**) TiN/IGTO IL (*t*_IGTO_ = 12 nm). (**f**–**j**) Output characteristics: (**f**) Without IL; (**g**) TiN IL; (**h**) TiN/IGTO IL (*t*_IGTO_ = 5 nm); (**i**) TiN/IGTO IL (*t*_IGTO_ = 8 nm); (**j**) TiN/IGTO IL (*t*_IGTO_ = 12 nm).

**Figure 8:**
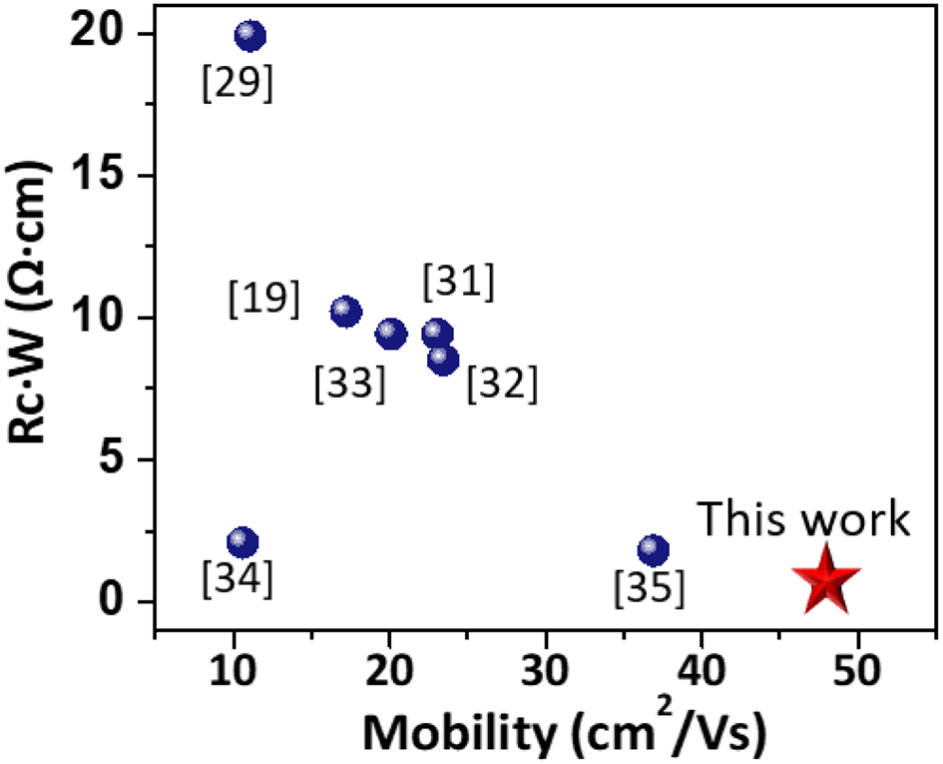
Comparison of *R*_C_*W* and *μ*_FE_ in oxide TFTs with different contact approaches.

Figure 7 shows electrical characteristics of IGZO TFTs with different IL structures. The IGZO TFTs without the IL exhibit device performances with *μ*_FE_ of 39.9 ± 1.6 cm^2^/Vs, current modulation ratio (*I*_ON/OFF_) > 10^8^, *SS* of 0.1 ± 0.03 V/dec, and *V*_TH_ of − 0.5 ± 0.4 V. The *μ*_FE_ is improved to 42.7 ± 1.4 cm^2^/Vs through insertion of a 3-nm-thick TiN IL, and it further increases using the TiN/IGTO multi-stack IL. Importantly, the *t*_IGTO_-dependent behavior is clearly observed, with the IGZO TFTs with *t*_IGTO_ of 8 nm exhibiting the highest *μ*_FE_ of 45.0 ± 1.6 cm^2^/Vs. Meanwhile, the devices with *t*_IGTO_ of 5 and 12 nm showed *μ*_FE_ values of 44.8 ± 1.3 and 42.4 ± 1.8 cm^2^/Vs, respectively. Figure S9 shows *V*_GS_-dependent *μ*_FE_ curves in the IGZO TFTs with different IL conditions. Such improvement by IL insertion is also seen in output characteristics (Fig. 7f–j). All device performances of the IGZO TFTs with different IL stacks are summarized in Table 1. Considering the significant impact of electrical contact on the electrical characteristics of the TFT, it is straightforward to understand the trend where the highest device performances are revealed in the IGZO TFTs with *t*_IGTO_ of 8 nm. This enhancement can be further pronounced in scaled oxide TFTs where the device performance is dictated by the electrical contact properties. Figure 8 and Table S1 are benchmarking graph and table, respectively. It is important to emphasize that the *ρ*_C_ (*R*_C_*W*) of 9.0 × 10^−6^ Ω·cm^2^ (0.7 Ω·cm) achieved in this study surpasses the state-of-the-art results reported in the literature for all types of multicomponent metal oxide transistors, demonstrating the superiority of this study.

**Table 1:** Summarized electrical figures of merit of IGZO TFTs with different IL stacks.

	*μ*_FE_ (cm^2^/Vs)	*SS* (V/dec)	*V*_TH_ (V)	*ρ*_C_ (Ω·cm^2^)	*R*_C_*W* (Ω·cm)
W/O IL	39.9 ± 1.6	0.1 ± 0.03	–0.5 ± 0.4	8.0 × 10^−4^	6.9
TiN IL	42.7 ± 1.4	0.1 ± 0.05	–0.9 ± 0.5	5.6 × 10^−5^	1.8
TiN/IGTO IL (*t*_IGTO_ = 5 nm)	44.8 ± 1.3	0.1 ± 0.03	–0.7 ± 0.4	1.4 × 10^−5^	0.9
TiN/IGTO IL (*t*_IGTO_ = 8 nm)	45.0 ± 1.6	0.1 ± 0.02	–0.6 ± 0.6	9.0 × 10^−6^	0.7
TiN/IGTO IL (*t*_IGTO_ = 12 nm)	42.4 ± 1.8	0.1 ± 0.05	–0.8 ± 0.6	3.0 × 10^−5^	1.3

## Conclusion

This study demonstrates a significant improvement in electrical contact properties of IGZO TFTs through insertion of a TiN/IGTO IL. The electrical contact properties are dependent on *t*_IGTO_, which is attributed to crystallographic change from the amorphous structure to the random polycrystalline structure of IGTO. Despite a decrease in bulk *ρ*_IGTO_ with increasing *t*_IGTO_, crystallization at *t*_IGTO_ of 12 nm aggravates the electrical contact due to side effects such as grain boundary infiltration and degraded MS interfaces. Therefore, the use of an amorphous 8-nm-thick IGTO thin-film is more effective in improving the electrical contact properties. As a result, *ρ*_C_ (*R*_C_*W*) decreases from 8.0 × 10^−4^ (6.9) to 9.0 × 10^−6^ Ω·cm^2^ (0.7 Ω·cm) and *μ*_FE_ increases from 39.9 to 45.0 cm^2^/Vs, compared to the IGZO devices without the IL. This study not only highlights the effectiveness of the multi-stack IL approach in enhancing electrical contact properties, but also demonstrates that the effect can be maximized using a highly conductive amorphous IL. Importantly, this contact scheme can be applied to various types of oxide TFTs, not just IGZO TFTs.

### Supplementary Information

Supplementary Information.

## Data Availability

The datasets used and/or analysed during the current study available from the corresponding author on reasonable request. Correspondence and requests for materials should be addressed to J.K.J. or T. K. (email: jkjeong1@hanyang.ac.kr; tkim13@kist.re.kr).
