# Supplementary material for: Specific contact resistivity reduction in amorphous IGZO thin-film transistors through a TiN/IGTO heterogeneous interlayer
Source: Sci Rep. 2024 May 13;14:10953. doi: 10.1038/s41598-024-61837-2 (PMC11091091; doi:10.1038/s41598-024-61837-2)
Supplement: Supplementary file 1 — Supplementary Information. [file 41598_2024_61837_MOESM1_ESM.docx]

**Supplementary Information**

**Specific contact resistivity reduction in amorphous IGZO thin-film transistors through a TiN/IGTO heterogeneous interlayer**

Joo Hee Jeong,^1^ Seung Wan Seo,^1^ Dongseon Kim,^1^ Seong Hun Yoon,^1^ Seung Hee Lee,^2^ Bong Jin Kuh,^2^ Taikyu Kim,^3,*^ and Jae Kyeong Jeong^1,*^

^1^Department of Electronic Engineering, Hanyang University, Seoul 04763, Republic of Korea

^2^Semiconductor R&D Center, Samsung Electronics Co., Hwaseong-si 18448 Gyeonggi-do, Republic of Korea

^3^Electronic Materials Research Center, Korea Institute of Science and Technology, Seoul 02792, Republic of Korea

*Address correspondence to J. K. Jeong (jkjeong1@hanyang.ac.kr), T. Kim (tkim13@kist.re.kr)

1. CONTACT SCHEME-DEPENDENT *ρ*_C_ AND *R*_C_*W*


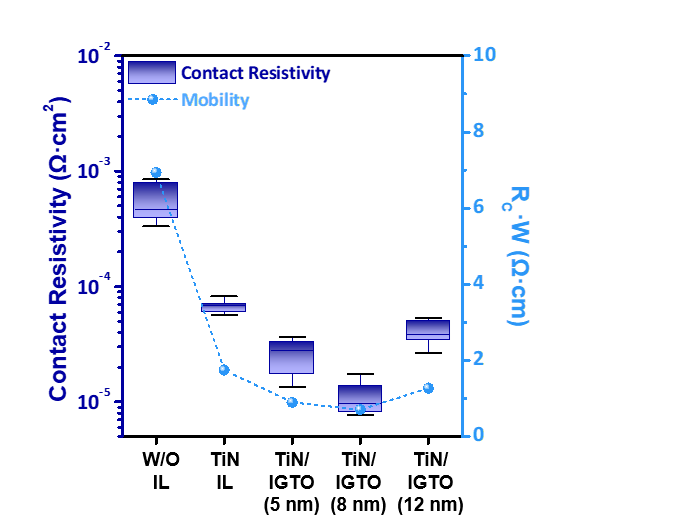


**Figure S1.** Contact scheme-dependent *ρ*_C_ and R_C_W in a-IGZO TFTs with different IL conditions: Without IL; 3-nm-thick TiN IL; 3/5-nm-thick TiN/IGTO IL; 3/8-nm-thick TiN/IGTO IL; 3/12-nm-thick TiN/IGTO IL.


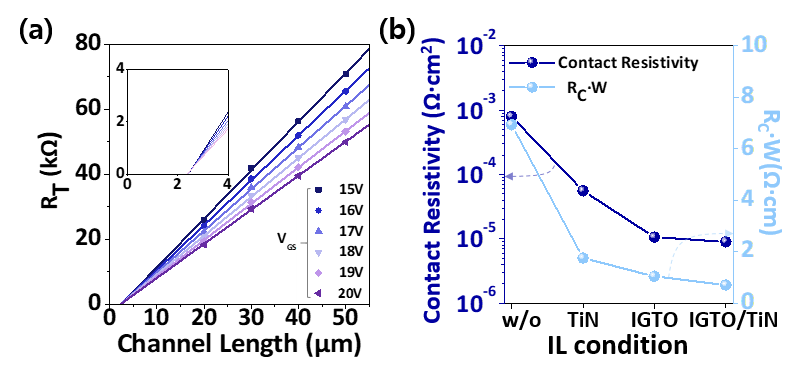


**Figure S2.** (a) V_GS_-dependent R_T_ variations of a IGZO TLM device with an 8-nm-thick IGTO IL at V_DS_ of 0.1 V. (b) Comparison of electrical contact properties of IGZO TLM devices using different ILs.

1. ANNEALING TEMPERATURE-DEPENDENT *ρ*_C_ AND *R*_C_*W*


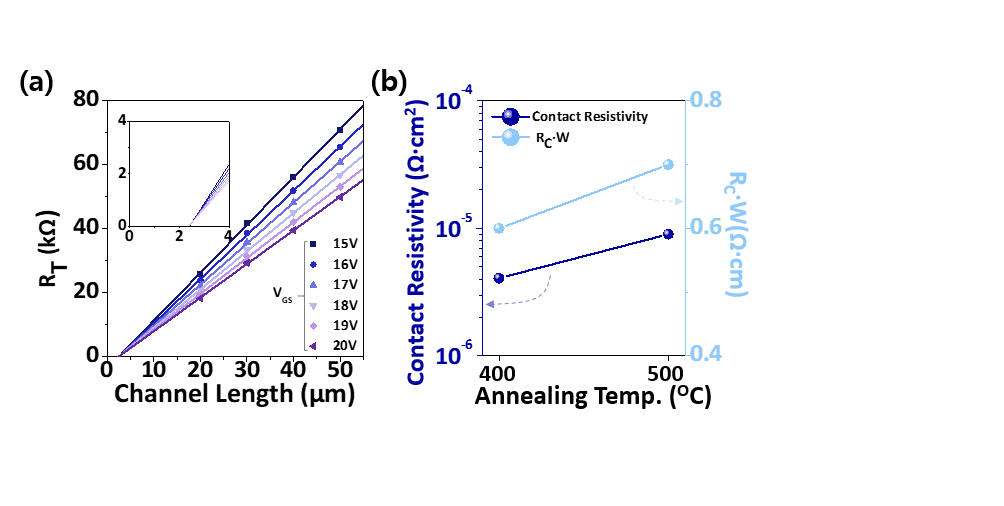


Figure S3. (a) V_GS_-dependent R_T_ variations of a 400 ^°^C-annealed IGZO TLM device with a 3-/8-nm-thick TiN/IGTO IL at V_DS_ of 0.1 V. (b) Comparison of electrical contact properties of IGZO TLM devices using the 3-/8-nm-thick TiN/IGTO IL annealed at 400 and 500 ^°^C.

1. UPS DEPTH PROFILE


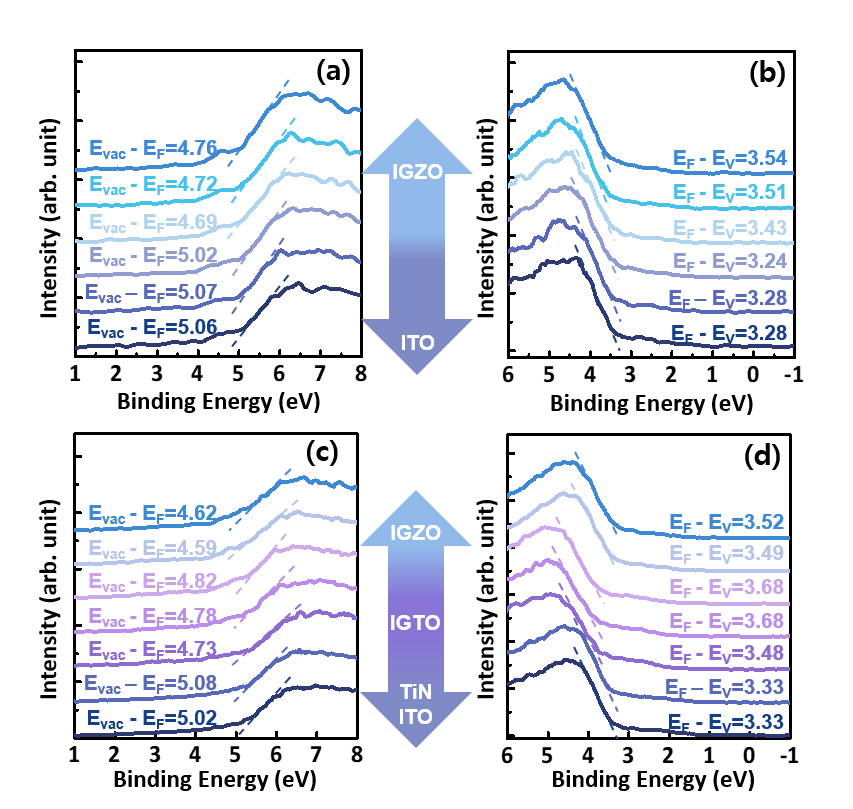


Figure S4. (a,c) Depth resolved work-function spectra: (a) ITO/IGZO; (c) ITO/TiN/IGTO/IGZO. (b,d) Depth resolved valence band spectra: (b) ITO/IGZO; (d) ITO/TiN/IGTO/IGZO.

1. CROSS-SECTIONAL HRTEM IMAGES


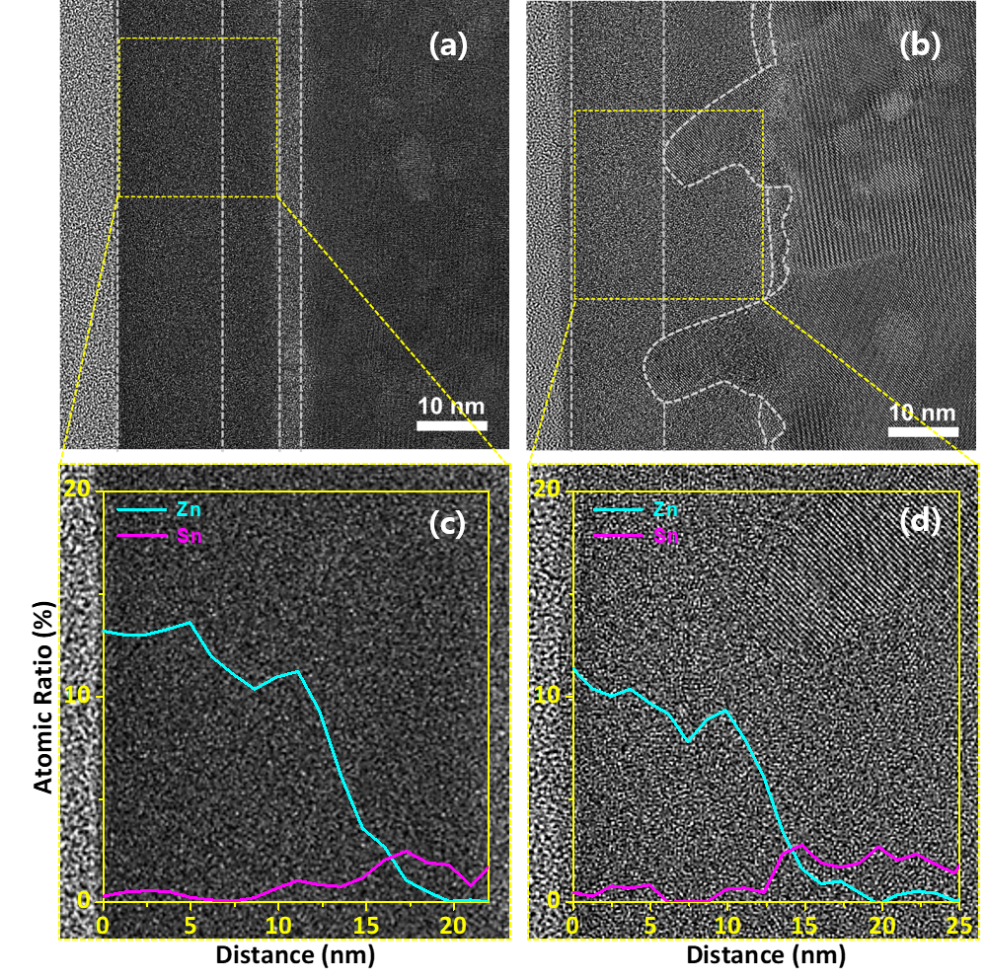


Figure S5. (a,b) Cross-sectional HRTEM images of ITO/TiN/IGTO/a-IGZO stacks with different *t*_IGTO_: (a) 8 nm; (b) 12 nm. (c,d) EDS depth profile for the corresponding thin-film stacks with different *t*_IGTO_: (c) 8 nm; (d) 12 nm.


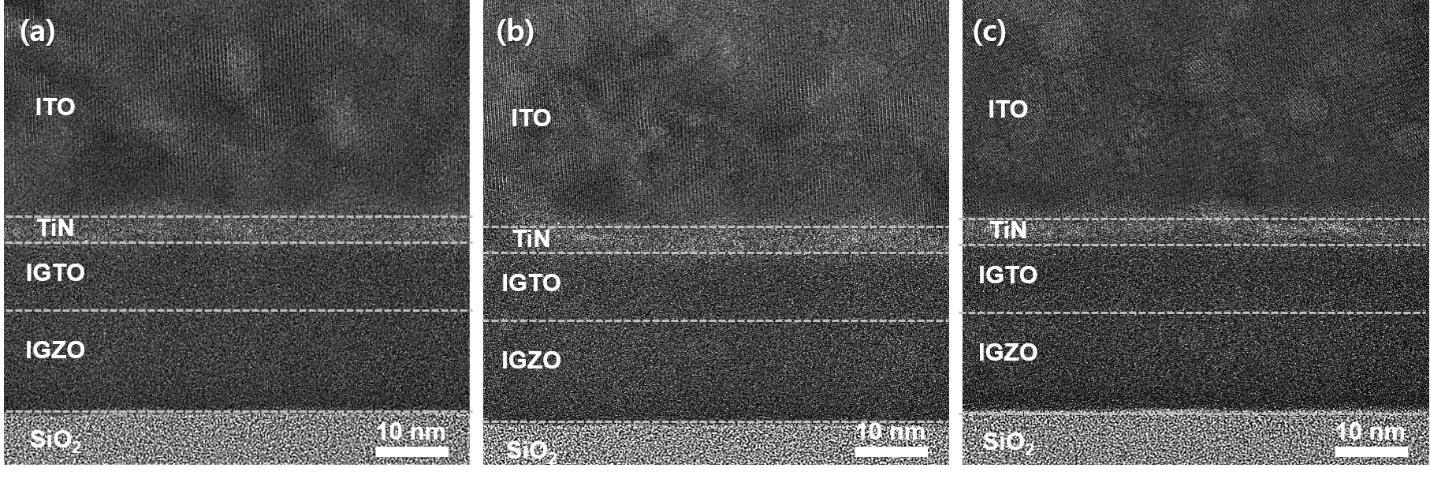


Figure S6. Cross-sectional HRTEM images of ITO/TiN/IGTO(8 nm)/a-IGZO stacks.


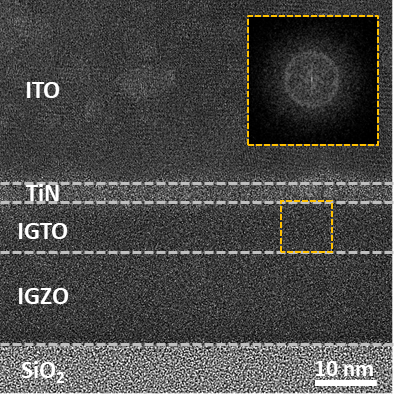


Figure S7. Cross-sectional HRTEM image of ITO/TiN (3 nm)/IGTO (8 nm)/a-IGZO stack. The inset is a fast Fourier Transform (FFT) result indicating that the IGTO has an amorphous structure.


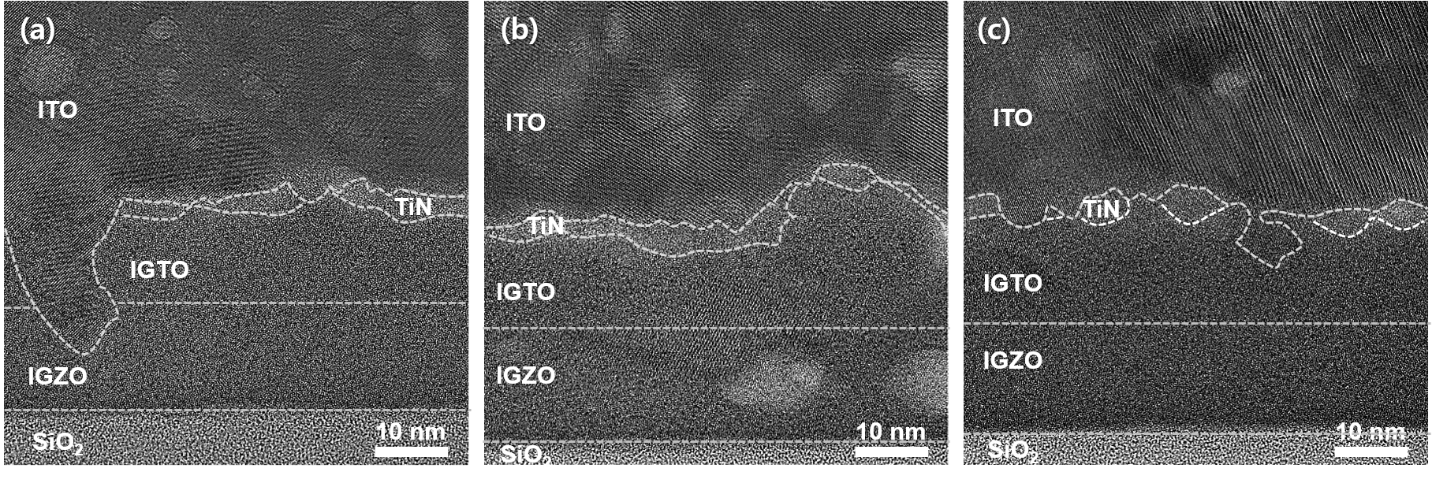


Figure S8. Cross-sectional HRTEM images of ITO/TiN/IGTO(12 nm)/a-IGZO stacks.

1. COMPARISION OF CONTACT SCHEME-DEPENDENT *μ*_FE_ AS A FUNCTION OF *V*_GS_


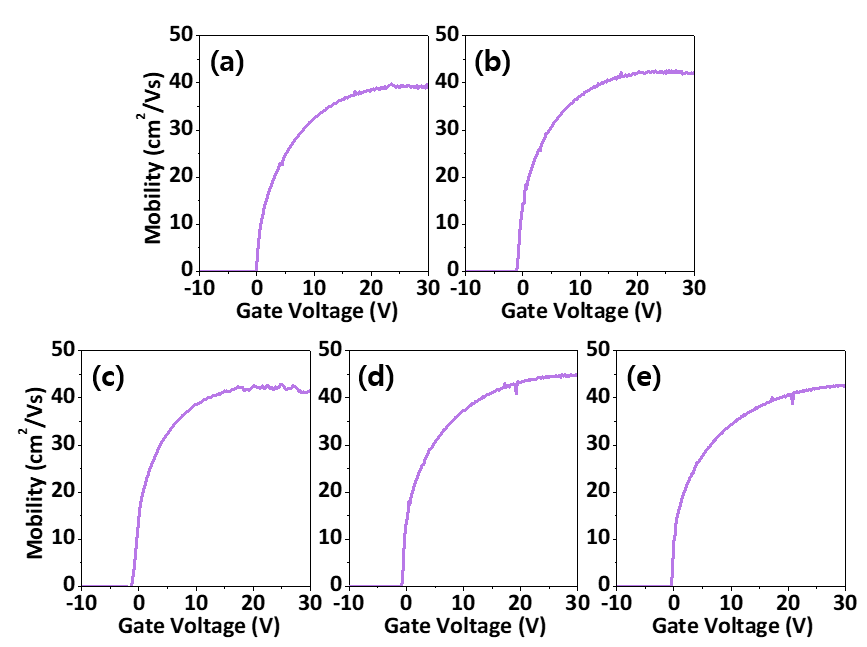


Figure S9. V_GS_ dependent *µ*_FE_ curves in the a-IGZO TFTs with different IL conditions; (a) without (W/O) IL, (b) 3-nm-thick TiN IL, (c) 3-/5-nm-thick TiN/IGTO IL, (d) 3-/8-nm-thick TiN/IGTO IL, (e) 3-/12-nm-thick TiN/IGTO IL.

1. BENCHMARKING TABLES

Table S1. Comparison of electrical figures of merit in oxide TFTs with different contact approaches.

| Approach | *Rc·W*  (Ω·cm) | *ρ*_C_  *(*Ω·cm^2^*)* | *μ*_FE_  (cm^2^/Vs) | Channel | Deposition method | Reference |
| --- | --- | --- | --- | --- | --- | --- |
| IGTO/TiN IL | 0.7 | 9.0 × 10^-6^ | 48.0 | IGZO | ALD | This work |
| ALD-Al_2_O_3_ ILD | 8.5 | 2.9 × 10^-4^ | 23.4 | IGZO | ALD | 2023 [32] |
| H plasma | - | 1.3 × 10^-6^ | - | IGZO | Sputter | 2022 [21] |
| B implantation | 10.2 | - | 17.2 | IGZO | Sputter | 2022 [19] |
| Cation composition | 2.1 | - | 10.6 | IGTO | ALD | 2022 [34] |
| Cation composition | 1.8 | - | 36.9 | IGZO | ALD | 2022 [35] |
| UV irradiation | 9.4 | - | 20.1 | IGZO | ALD | 2021 [33] |
| Ar plasma | 19.9 | - | 11.1 | IGZO | Sputter | 2016 [29] |
| Metal oxidation | - | 8.7 × 10^-3^ | 8.5 | IGZO | Sputter | 2013 [27] |
| H_2_ plasma | - | 9.9 × 10^-5^ | - | IGZO | Sputter | 2012 [22] |
| Metal oxidation | - | 1.2 × 10^-4^ | 10.8 | IGZO | Sputter | 2011 [26] |
